# Possible role of insulin resistance in activation of plasma xanthine oxidoreductase in health check-up examinees

**DOI:** 10.1038/s41598-022-11094-y

**Published:** 2022-06-18

**Authors:** Masafumi Kurajoh, Shinya Fukumoto, Seigo Akari, Takayo Murase, Takashi Nakamura, Kanae Takahashi, Hisako Yoshida, Shinya Nakatani, Akihiro Tsuda, Tomoaki Morioka, Katsuhito Mori, Yasuo Imanishi, Kazuto Hirata, Masanori Emoto

**Affiliations:** 1 Department of Metabolism, Endocrinology, and Molecular Medicine, Osaka City University Graduate School of Medicine, Osaka, Japan; 2 Department of Premier Preventive Medicine, Osaka City University Graduate School of Medicine, 1-4-3, Asahi-machi, Abeno-ku, Osaka, 545-8585 Japan; 3 Department of Research and Development, Sanwa Kagaku Kenkyusho Co., Ltd., Aichi, Japan; 4 Department of Medical Statistics, Osaka City University Graduate School of Medicine, Osaka, Japan; 5 Department of Nephrology, Osaka City University Graduate School of Medicine, Osaka, Japan; 6 Osaka City University, Osaka, Japan

**Keywords:** Biochemistry, Metabolic syndrome, Obesity

## Abstract

We previously found an association of insulin resistance (IR) with plasma xanthine oxidoreductase (XOR) activity in a cross-sectional study. However, whether IR induces increased XOR activity has not been elucidated. This retrospective longitudinal observational study included 347 participants (173 males, 174 females) who underwent annual health examinations and were medication naïve. Homeostasis model assessment of IR (HOMA-IR) index, and physical and laboratory measurements were determined at the baseline. At baseline and 12-month follow-up examinations, plasma XOR activity was determined using our novel assay based on [^13^C_2_,^15^N_2_] xanthine and liquid chromatography/triple quadrupole mass spectrometry. Subjects with IR, defined as HOMA-IR index ≥ 1.7 (n = 92), exhibited significantly (*p* < 0.001) higher plasma XOR activity levels than those without IR (n = 255), with an increase in that activity seen in 180 (51.9%) after 12 months. Multivariable linear and logistic regression analyses showed that IR, but not BMI or waist circumference, at baseline was significantly associated with plasma XOR activity (β = 0.094, *p* = 0.033) and increased plasma XOR activity over the 12-month period (odds ratio, 1.986; 95% confidence interval, 1.048–3.761; *p* = 0.035), after adjustments for various clinical parameters, including plasma XOR activity at baseline. These results suggest that IR induces increased plasma XOR activity in a manner independent of adiposity.

## Introduction

Hyperuricemia is frequently observed in individuals with obesity, who are also characterized by insulin resistance (IR)^1,2^. Studies of subjects with obesity have shown that hyperinsulinemia induced by IR can cause hyperuricemia by reducing renal excretion of uric acid through its increased renal reabsorption^3^. On the other hand, several reports have emphasized that such individuals also have increased production of uric acid^1,4^, though the underling mechanisms remain unclear.

Regarding the mechanisms of increased uric acid production, Nagao and colleagues suggested that secretion of hypoxanthine, produced by adipose tissue, is increased in individuals with obesity, with circulating hypoxanthine taken up by the liver and catabolized to uric acid, resulting in increased uric acid production^5^. In addition, a recent study found a positive association between body mass index (BMI) and plasma xanthine oxidoreductase (XOR) activity^6^. XOR, mainly expressed in the liver and intestines in humans^5,7–9^, is a rate-limiting enzyme that catalyzes oxidation, not only from hypoxanthine to xanthine but also from xanthine to uric acid, in the purine metabolism pathway^10^. While the regulatory mechanisms of XOR activity have not been fully clarified, hepatic, though not intestinal XOR activity, is known to be increased in gouty patients with over-production of uric acid^11,12^, suggesting the existence of an XOR activity regulatory mechanism.

One of the reasons for lack of research regarding regulation of XOR activity and its pathological significance is lack of an accurate method to assess that activity in humans. Until recently, circulating XOR activity as well could not be accurately measured, since the level of activity is extremely lower in humans as compared to that in rodents^13^. However, we recently developed a highly sensitive and accurate method for determining plasma XOR activity in humans that utilizes an assay of stable isotope-labeled [^13^C_2_,^15^N_2_] xanthine with liquid chromatography (LC)/triple quadrupole mass spectrometry (TQMS)^14,15^. Using that method, our previous cross-sectional and longitudinal studies revealed that plasma XOR activity is independently associated with serum uric acid level^16–18^, suggesting that plasma XOR activity reflects XOR activity in the liver and that serum uric acid level is regulated by that activity. Furthermore, we reported in a cross-sectional study that IR was significantly associated with plasma XOR activity^19^. Taken together, it is speculated that IR contributes to increased uric acid production by stimulating intrahepatic XOR activity in individuals with obesity characterized by IR.

To the best of our knowledge, no longitudinal investigations have been performed to examine the predictive association of IR with XOR activity. In the present retrospective longitudinal observational study, we examined plasma XOR activity related to IR after 12 months in medication-naïve subjects who underwent annual health examinations using our novel XOR assay to investigate whether IR predicts and induces increased XOR activity.

## Results

### Clinical characteristics of subjects, and comparisons between those with and without IR

The characteristics of the enrolled subjects (n = 347) are presented in Table 1. The median values for plasma XOR activity, BMI, and waist circumference were 32.1 pmol/h/mL, 22.5 kg/m^2^, and 81.5 cm, respectively. Subjects with IR (n = 92) had significantly (*p* < 0.001) higher levels of plasma XOR activity (47.6 vs. 27.4 pmol/h/mL), BMI (25.0 vs. 21.6 kg/m^2^), and waist circumference (87.8 vs. 79.1 cm), as well as of alanine aminotransferase (ALT), fasting plasma glucose (FPG), glycated hemoglobin (HbA1c), and uric acid, as compared to those without IR (n = 255) (Table 2).

**Table 1:** Clinical characteristics of subjects (n = 347) at baseline and 12-month examinations.

	Baseline	12-month
Age, years	47.0 (41.0–53.0)	
Males, n	173 (49.9)	
Alcohol drinking habit, n	145 (41.8)	151 (43.5)
Smoking habit, n	55 (15.9)	52 (15.0)
BMI, kg/m^2^	22.5 (20.5–24.5)	22.7 (20.6–24.8)
Waist circumference, cm	81.5 (76.4–87.5)	82.5 (76.4–88.5)
AST, U/L	19.0 (16.0–23.0)	19.0 (16.0–23.0)
ALT, U/L	17.0 (12.0–23.0)	16.0 (13.0–23.0)
eGFR, mL/min/1.73 m^2^	80.9 (72.5–89.9)	78.6 (70.9–85.8)
FPG, mg/dL	99.0 (94.0–105.0)	99.0 (94.0–105.0)
HbA1c, %	5.6 (5.4–5.8)	5.6 (5.4–5.8)
Uric acid, mg/dL	5.2 (4.3–6.1)	5.2 (4.3–6.1)
HOMA-IR	1.2 (0.9–1.7)	1.1 (0.8–1.7)
Plasma XOR activity, pmol/h/mL	32.1 (19.8–56.0)	33.0 (20.5–54.5)

Values are expressed as median (interquartile range) or number (%).

*BMI* body mass index, *AST* aspartate aminotransferase, *ALT* alanine aminotransferase, *eGFR* estimated glomerular filtration rate, *FPG* fasting plasma glucose, *HbA1c* glycated hemoglobin, *HOMA-IR* homeostatic model assessment of insulin resistance, *XOR* xanthine oxidoreductase.

**Table 2:** Comparison of clinical characteristics between subjects with and without IR at baseline.

	With IR (n = 92)	Without IR (n = 255)	*p*
Age, years	48.0 (42.0–54.0)	46.0 (41.0–53.0)	0.406
Males, n	47 (51.1)	126 (49.4)	0.783
Alcohol drinking habit, n	38 (41.3)	107 (42.0)	0.913
Smoking habit, n	15 (16.3)	40 (15.7)	0.889
BMI, kg/m^2^	25.0 (23.2–27.0)	21.6 (20.0–23.4)	< 0.001
Waist circumference, cm	87.8 (82.1–94.0)	79.1 (74.0–84.5)	< 0.001
AST, U/L	20.0 (17.0–23.8)	19.0 (16.0–23.0)	0.101
ALT, U/L	22.0 (16.0–32.8)	16.0 (11.0–20.0)	< 0.001
eGFR, mL/min/1.73 m^2^	82.5 (72.8–91.4)	79.7 (72.4–89.6)	0.342
FPG, mg/dL	104.0 (99.0–109.0)	96.0 (93.0–103.0)	< 0.001
HbA1c, %	5.7 (5.5–5.9)	5.6 (5.4–5.7)	< 0.001
Uric acid, mg/dL	5.5 (4.5–6.5)	4.8 (4.0–5.9)	0.002
HOMA-IR	2.3 (2.0–2.8)	1.0 (0.7–1.3)	< 0.001
Plasma XOR activity, pmol/h/mL	47.6 (30.1–95.0)	27.4 (17.7–48.7)	< 0.001

Values are expressed as median (interquartile range) or number (%). *p* Values are shown for comparisons between subjects with (HOMA-IR ≥ 1.7) and without (HOMA-IR < 1.7) IR.

*IR* insulin resistance, *BMI* body mass index, *AST* aspartate aminotransferase, *ALT* alanine aminotransferase, *eGFR* estimated glomerular filtration rate, *FPG* fasting plasma glucose, *HbA1c* glycated hemoglobin, *HOMA-IR* homeostatic model assessment of insulin resistance, *XOR* xanthine oxidoreductase.

### Plasma XOR activity after 12 months

The characteristics of the subjects after 12 months are presented in Table 1. The median value for plasma XOR activity was 33.0 pmol/h/mL. As compared to the baseline, 180 (51.9%) of the subjects showed increased and 167 (48.1%) showed no increase in plasma XOR activity after 12 months. Furthermore, as compared to the non-increased plasma XOR activity group, aspartate aminotransferase, ALT, estimated glomerular filtration rate (eGFR), and plasma XOR activity at the baseline were significantly lower in the increased plasma XOR activity group (Supplementary Table S1).

### Association of IR with plasma XOR activity after 12 months

To examine whether the presence of IR at the baseline was associated with plasma XOR activity at the 12-month examination, multivariable linear regression analyses were performed (Table 3). Plasma XOR activity at the baseline was significantly associated with that activity after 12 months (β = 0.604, *p* < 0.001). Furthermore, presence of IR, but not BMI or waist circumference at the baseline, was significantly associated with plasma XOR activity at the 12-month follow-up examination (β = 0.094, *p* = 0.033). The “sex*IR” interaction was not significant (*p* = 0.740), providing no evidence that sex has an effect to modify the relationship of IR at baseline with plasma XOR activity after 12 months. Additionally, age, sex, alcohol drinking habit, smoking habit, ALT, HbA1c, and eGFR each showed no significant association with plasma XOR activity at the 12-month examination.

**Table 3:** Multivariable linear regression analysis of possible factors associated with plasma XOR activity at 12-month follow-up examination.

Explanatory variables	β	*p*
Age	− 0.007	0.872
Sex (male = 1, female = 0)	0.077	0.071
Alcohol drinking habit (present = 1, absent = 0)	0.008	0.825
Smoking habit (present = 1, absent = 0)	0.009	0.811
ALT	0.061	0.250
HbA1c	0.066	0.114
eGFR	− 0.056	0.175
BMI	0.004	0.960
Waist circumference	0.013	0.857
IR (present = 1, absent = 0)	0.094	0.033
Plasma XOR activity (at baseline)	0.604	< 0.001
Adjusted R^2^/*p*	0.546/ < 0.001	

Values shown represent standardized partial regression coefficient (β values) and level of significance.

*ALT* alanine aminotransferase, *HbA1c* glycated hemoglobin, *eGFR* estimated glomerular filtration rate, *BMI* body mass index, *IR* insulin resistance, *XOR* xanthine oxidoreductase.

### Association of IR with increase in plasma XOR activity over 12 months

To further examine whether the presence of IR at the baseline was independently associated with an increase in plasma XOR activity over the 12-month period, multivariable logistic regression analysis was performed (Table 4). IR, but not BMI or waist circumference, at the baseline was significantly and independently associated with increased plasma XOR activity at the 12-month examination (odds ratio, 1.986; 95% confidence interval, 1.048–3.761; *p* = 0.035). The “sex*IR” interaction was not significant (*p* = 0.592), again providing no evidence that sex has an effect to modify the relationship of IR at the baseline with an increase in plasma XOR activity after 12 months.

**Table 4:** Multivariable logistic regression analysis regarding increase in plasma XOR activity over 12-month period.

Explanatory variables	OR (95% CI)	*p*
BMI	0.944 (0.811–1.100)	0.461
Waist circumference	1.015 (0.960–1.073)	0.600
IR (present = 1, absent = 0)	1.986 (1.048–3.761)	0.035

The multivariable logistic regression model used for analysis of increase in plasma XOR activity over a 12-month period included age, sex, alcohol drinking habit, smoking habit, ALT, HbA1c, eGFR, and plasma XOR activity, as well as BMI, waist circumference, and presence of IR at baseline as covariates.

*BMI* body mass index, *IR* insulin resistance, *ALT* alanine aminotransferase, *HbA1c* glycated hemoglobin, *eGFR* estimated glomerular filtration rate, *XOR* xanthine oxidoreductase, *OR* odds ratio, *CI* confidence interval.

## Discussion

This is the first known study to investigate the direct association of IR with plasma XOR activity. Our findings showed that IR, but not BMI or waist circumference, at the baseline predicted plasma XOR activity (Table 3) as well as an increase in that activity (Table 4) after 12 months in subjects who underwent annual health examinations. These results indicate that IR as result of obesity might induce increased plasma XOR activity.

Previous studies including ours have found significant associations between IR and plasma XOR activity in young healthy subjects^6^ and general populations^19,20^, as well as subjects with type 2 diabetes mellitus and metabolic syndrome^21^. However, those findings were based on cross-sectional study results. Recently, Furuhashi and colleagues conducted a longitudinal study of a general population, and presented findings showing no significant association between annual changes in model assessment of IR (HOMA-IR) index and plasma XOR activity^22^. However, they used only simple regression analysis and did not perform adjustments for the effects of multiple factors. To date, no other studies have examined whether IR is useful to predict future plasma XOR activity, while the present is the first to show a predictive association of IR with increased plasma XOR activity (Tables 3, 4). Thus, we concluded that IR might induce an increase in XOR activity.

HOMA-IR, calculated using fasting insulin and glucose levels, has been well established as a simple and excellent index for IR^23–25^. A HOMA-IR value ≥ 2.5 is considered to be a reasonable threshold for IR, as the ‘health-associated’ reference interval for HOMA-IR, shown to cover the central 95% of 2153 healthy Japanese subjects, was established as between 0.4 and 2.4 by applying the stringent Clinical and Laboratory Standards Institute C28-A3 document^26^. On the other hand, HOMA-IR ≥ 1.7 is also considered to be a threshold value for IR, since the ‘decision-based’ limit for HOMA-IR found to indicate subjects with a high risk for metabolic syndrome in at study of 6868 non-diabetic Japanese subjects was 1.7^27^. In the present study, the association of IR with XOR activity, thought to be the cause of hyperuricemia, a condition closely related to metabolic syndrome, was examined. Therefore, HOMA-IR ≥ 1.7 was used as the threshold value for IR.

Despite the impact of IR on XOR activity, it remains unclear how it induces an increase in that activity. IR is considered to increase de novo purine synthesis through activation of the pentose phosphate pathway^28^. Furthermore, purine intermediate metabolites, such as inosine, hypoxanthine, and xanthine, have been reported to increase hepatic XOR activity in chicks, mice, and humans as an adaptation to elevated concentrations of purine intermediate metabolites^11,29–31^. This framework might be a mechanism for increased XOR activity caused by IR. Furthermore, IR-induced inflammatory cytokines might also lead to increased XOR activity. Recently, hyperinsulinemia, characteristic of IR, was reported to decrease M2a-subtype macrophage activation via insulin receptor substrate-2 downregulation, leading to increases in inflammatory cytokines in the liver^32^. Inflammatory cytokines such as interferon-gamma, tumor necrosis factor-alpha, and interleukin-1 and -6 have been shown to increase XOR activity in the liver, as well as mammary, renal, and pulmonary epithelial cells by increasing XOR gene expression at the transcriptional level^33–36^. Thus, IR might induce XOR activation via inflammatory cytokines. However, purine intermediate metabolites and inflammatory cytokines were not measured in the present examinations, thus it was not possible to investigate whether purine intermediate metabolites and/or inflammatory cytokines mediate the association between IR and XOR activity. The mechanisms related to XOR activation induced by IR require further investigation.

Over the present 12-month examination period, BMI and waist circumference showed no significant association with plasma XOR activity or increase in that activity (Tables 3, 4). These results are consistent with those obtained in our previous cross-sectional study showing that BMI was not significantly associated with plasma XOR activity^19^. In mice, XOR is highly expressed in adipose tissue as well as the liver and intestines, and uric acid is secreted from adipose tissue^37^, whereas in humans, XOR is known to be expressed in the liver and intestines, but not adipose tissue^5,7–9^. Therefore, our previous cross-sectional^19^ and present longitudinal results can be explained by localization of XOR in humans. However, subjects with obesity are reported to have increased production of uric acid in addition to decreased urinary excretion of uric acid^1,4^. On the other hand, IR has been shown capable to predict development of hyperuricemia independent of the degree of obesity^38,39^. Interestingly, administration of metformin or troglitazone, each of which improves IR in liver and muscle tissues^40,41^, was previously found to decrease serum uric acid level in a manner unrelated to renal uric acid excretion^42,43^. Furthermore, metformin has also been reported to cause a decrease in plasma XOR activity^44^. Although the mechanisms underlying increased production of uric acid in obese individuals have not been fully clarified, the present results along with those presented in prior studies suggest that IR contributes to an increase in uric acid production by stimulating XOR activity in the liver of those affected by obesity.

An important limitation of this study is that few subjects with obesity (3.2%), defined as BMI ≥ 30 kg/m^2^^45^, and IR (9.8%), defined as HOMA-IR ≥ 2.5^26^, were included. Since individuals who were receiving no medication were enrolled in order to eliminate the impact of therapeutic drugs, patients with obesity and IR, based on the criteria described above, were less likely to be included in the study population because they are often treated with medication for lifestyle-related diseases, such as diabetes, hypertension, and dyslipidemia. Therefore, sub-analysis of obese subjects could not be fully performed, nor could we conduct a full investigation of the association between IR defined as HOMA-IR ≥ 2.5 and XOR activity. However, by not including subjects with severe obesity, we considered that examination of the direct relationship between IR and XOR activity independent of obesity was more easily performed. Since accumulated visceral fat causes not only IR but is also known to secrete purine intermediate metabolites that may have effects on the relative activation of XOR^11,29–31^, its impact may be stronger in individuals with severely obesity. Furthermore, the present results also suggest that XOR activation begins in a mildly obese state. Another limitation is that neither waist-to-hip ratio or visceral fat area (VFA) was measured in the present cohort, which are known to be important indicators of obesity^46^. Although the present results show that IR can predict plasma XOR activity and an increase in that activity, independent of degree of obesity, based on BMI and waist circumference, we were not able to investigate its association with VFA or waist-to-hip ratio. For confirmation, it will be necessary to validate the present findings by investigating a larger population that includes individuals with moderate to severe obesity and IR, as well as measurements of VFA, waist-to-hip ratio, purine intermediate metabolites, and inflammatory cytokines.

In summary, the present investigation showed that IR at baseline predicts plasma XOR activity and an increase in that activity over a 12-month period in a manner independent of degree of obesity. The results obtained with our novel assay suggest that IR caused by obesity induces increased XOR activity, resulting in increased production of uric acid.

## Materials and methods

### Study design

The MedCity21 health examination registry was initiated in April 2015 in a comprehensive manner to elucidate the causes of various diseases occurring in adults, including cancer, diabetes mellitus, cardiovascular and cerebrovascular diseases, mental disorders, dyslipidemia, hypertension, hyperuricemia, and obesity, as well as chronic respiratory, liver, digestive, gynecological, and skin diseases, for development of advanced diagnostic techniques, along with treatment and prevention methods for affected patients^17,19,47–50^. Individuals who underwent medical examinations at MedCity21, an advanced medical center for preventive medicine established at Osaka City University Hospital (Osaka, Japan), were registered. The MedCity21 health examination registry protocol was approved by the Ethics Committee of Osaka City University Graduate School of Medicine (approval No. 2927). Written informed consent was obtained from all subjects and the study was conducted in full accordance with the Declaration of Helsinki. The present study protocol was approved by the Ethics Committee of Osaka City University Graduate School of Medicine (approval No. 3684) and performed with an opt-out option, explained in instructions on the website of the hospital.

### Participants

Using the MedCity21 health examination registry, we selected the final 350 sequential participants who did not receive any medical agent administration during the study and underwent medical examinations in both 2016 and 2017 with an interval of 12 months. For this analysis, participants with missing data regarding alcohol consumption (n = 1) or serum immunoreactive insulin (IRI) level (n = 2) were excluded. As a result, 347 participants (173 males, 174 females) were enrolled as subjects in the present retrospective longitudinal observational study.

### Physical and laboratory measurements at baseline

Information regarding height, body weight, and waist circumference, as well as smoking and alcohol consumption habits was obtained at the initial examination in 2016 and then again at the follow-up examination in 2017. Regarding alcohol drinking, the subjects were divided into those who answered almost never, 1–4 days/week, or 5–7 days/week for current intake. Those in the latter two groups were considered to have an alcohol drinking habit. BMI was calculated as weight in kilograms divided by the square of height in meters (kg/m^2^). Blood was drawn after an overnight fast. Biochemical parameters were analyzed using a standard laboratory method as part of the MedCity21 protocol and the remaining blood samples were stored at − 80 °C. eGFR was calculated using an equation designed for Japanese subjects, as previously described^51^. HbA1c levels were determined using high-performance liquid chromatography^52^.

### Definition of IR at baseline

Serum IRI level was determined with an electrochemiluminescence immunoassay (Roche Diagnostics K.K., Tokyo, Japan). The HOMA-IR index was calculated according to the following formula: fasting IRI (µU/mL) × FPG (mg/dL)/405^23–25^. Presence of IR was defined as HOMA-IR index ≥ 1.7, as previously described^27^.

### Plasma XOR activity at baseline and after 12 months

Plasma XOR activity was determined using freshly frozen samples obtained in 2016 and again after 12 months in 2017, which were maintained at − 80 °C until the time of the assay, performed with our recently established novel method for assays of stable isotope-labeled [^13^C_2_,^15^N_2_] xanthine with LC/TQMS at Mie Research Laboratories and Sanwa Kagaku Kenkyusho Co., Ltd, as previously described^14,15^. Briefly, 100-µL aliquots of plasma were purified using a Sephadex G25 column, then mixed with Tris buffer (pH 8.5) containing [^13^C_2_,^15^N_2_] xanthine as a substrate and nicotinamide adenine dinucleotide^+^, with [^13^C_3_,^15^N_3_] uric acid used as an internal standard, then the mixtures were incubated at 37 °C for 90 min. Subsequently, they were combined with methanol (500 µL) and centrifuged at 2000 ×*g* for 15 min at 4 °C. Supernatants were collected and transferred to new tubes, then dried using a centrifugal evaporator. The residues were reconstituted in 150 μL of distilled water and filtered through an ultrafiltration membrane, then measurements were performed using LC/TQMS. Calibration standard samples were examined for the amount of [^13^C_2_,^15^N_2_] uric acid produced, which was calculated by use of a calibration curve, with XOR activity expressed as the amount (pmol/h/mL). The inter- and intra-assay coefficients of variation of plasma XOR activity were 9.1% and 6.5%, respectively^14^. Increased and non-increased plasma XOR activity groups were defined based on results showing increased or decreased/unchanged plasma XOR activity over the 12-month period between examinations, since there is no reference value presently available.

### Statistical analysis

Values are expressed as number (%) or median. To compare variables between groups, Mann–Whitney’s U test (continuous variables) and a chi-squared test (categorical variables) were used. Plasma XOR activity was logarithmically transformed (log) to follow a normal distribution, before submitting to multivariable regression analysis. Multivariable linear analysis was performed to determine whether presence of IR at the baseline was independently associated with plasma XOR activity and logistic regression analysis was performed to determine whether IR at the baseline was independently associated with an increase in that activity over a 12-month period, after adjustments using various clinical parameters determined at the baseline, including BMI and waist circumference, as well as age, sex, alcohol drinking habit, smoking habit, ALT, HbA1c, eGFR, and plasma XOR activity. We did not select serum uric acid level as a covariate, because uric acid is produced from xanthine by an XOR-catalyzed reaction. In addition, we incorporated a two-factor interaction term (sex*presence of IR) into multivariable linear and logistic regression analyses in order to assess the effect of sex on the relationship of IR with plasma XOR activity as well as increase in plasma XOR activity over the 12-month period. All statistical analyses were carried out using the Statistical Package for the Social Sciences (IBM SPSS Statistics for windows, version 22.0; IBM Corp. Armonk, NY, USA). All reported p values are two-tailed and were considered to indicate statistical significance at *p* < 0.05.

## Supplementary Information

Supplementary Information.
