# Supplementary material for: Possible role of insulin resistance in activation of plasma xanthine oxidoreductase in health check-up examinees
Source: Sci Rep. 2022 Jun 18;12:10281. doi: 10.1038/s41598-022-11094-y (PMC9206666; doi:10.1038/s41598-022-11094-y)
Supplement: Supplementary file 1 — Supplementary Information. [file 41598_2022_11094_MOESM1_ESM.docx]

**Supplementary Table S1. Comparison of clinical characteristics at baseline between subjects with and without increase in plasma XOR activity over 12-month period**

|  |  | Increased  group (n=180) | Non-increased  group (n=167) |  | p |
| --- | --- | --- | --- | --- | --- |
| Age, years |  | 48.0 (41.0-53.0) | 46.0 (41.0-53.0) |  | 0.328 |
| Males, n |  | 86 (47.8) | 87 (52.1) |  | 0.422 |
| Alcohol drinking habit, n |  | 71 (39.4) | 74 (44.3) |  | 0.358 |
| Smoking habit, n |  | 24 (13.3) | 31 (18.6) |  | 0.183 |
| BMI, kg/m^2^ |  | 22.4 (20.3-24.0) | 22.8 (20.7-24.9) |  | 0.081 |
| Waist circumference, cm |  | 81.0 (76.0-87.0) | 82.4 (76.5-88.4) |  | 0.170 |
| AST, U/L |  | 18.0 (15.0-22.0) | 21.0 (17.0-24.0) |  | <0.001 |
| ALT, U/L |  | 16.0 (11.0-20.8) | 18.0 (14.0-25.0) |  | <0.001 |
| eGFR, mL/min/1.73 m^2^ |  | 78.2 (71.7-87.0) | 83.1 (73.4-91.6) |  | 0.004 |
| FPG, mg/dL |  | 98.5 (93.0-105.0) | 99.0 (94.0-105.0) |  | 0.436 |
| HbA1c, % |  | 5.6 (5.4-5.8) | 5.6 (5.4-5.7) |  | 0.282 |
| Uric acid, mg/dL |  | 5.0 (4.2-6.0) | 5.1 (4.0-6.1) |  | 0.653 |
| HOMA-IR |  | 1.2 (0.8-1.8) | 1.2 (0.9-1.7) |  | 0.564 |
| Plasma XOR activity, pmol/h/mL |  | 25.9 (16.3-42.4) | 43.3 (26.3-73.7) |  | <0.001 |

Values are expressed as median (interquartile range) or number (%). P values are shown for comparisons between the increased and non-increased plasma XOR activity groups.

Abbreviations: XOR, xanthine oxidoreductase; BMI, body mass index; AST, aspartate aminotransferase; ALT, alanine aminotransferase; eGFR, estimated glomerular filtration rate; FPG, fasting plasma glucose; HbA1c, glycated hemoglobin; HOMA-IR, homeostatic model assessment of insulin resistance
